# Characteristics of head frequency response in blunt impacts: a biomechanical modeling study

**DOI:** 10.3389/fbioe.2024.1364741

**Published:** 2024-02-26

**Authors:** Guibing Li, Shengkang Xu, Tao Xiong, Kui Li, Jinlong Qiu

**Affiliations:** ^1^ School of Mechanical Engineering, Hunan University of Science and Technology, Xiangtan, China; ^2^ School of Pharmacy and Bioengineering, Chongqing University of Technology, Chongqing, China; ^3^ College of Medical Informatics, Chongqing Medical University, Chongqing, China; ^4^ Chongqing Key Laboratory of Vehicle/Biological Crash Safety, Chongqing, China; ^5^ Institute of Traffic Medicine, Army Military Medical University, Chongqing, China

**Keywords:** traumatic brain injury, head frequency response, blunt impact, finite element modeling, wavelet packet

## Abstract

Existing evaluation criteria for head impact injuries are typically based on time-domain features, and less attention has been paid to head frequency responses for head impact injury assessment. The purpose of the current study is, therefore, to understand the characteristics of human body head frequency response in blunt impacts via finite element (FE) modeling and the wavelet packet analysis method. FE simulation results show that head frequency response in blunt impacts could be affected by the impact boundary condition. The head energy peak and its frequency increase with the increase in impact; a stiffer impact block is associated with a higher head energy peak, and a bigger impact block could result in a high proportion of the energy peak. Regression analysis indicates that only the head energy peak has a high correlation with exiting head injury criteria, which implies that the amplitude–frequency aggregation characteristic but not the frequency itself of the head acceleration response has predictability for head impact injury in blunt impacts. The findings of the current study may provide additional criteria for head impact injury evaluation and new ideas for head impact injury protection.

## 1 Introduction

Head impact injury is a major public health issue, and the prevention and treatment of such injuries have become a significant challenge in the medical field globally, as they have a severe impact on the human body, not only leading to varying degrees of behavioral and functional disorders but also having a high fatality rate (MacDonald et al., 2014; Popescu C et al., 2015). There are many sources of head impact injuries, including falls, traffic accidents, dropped objects from heights, and sports such as football and boxing, among which traffic accidents are the dominant source of head impact injuries (Organization, 2018).

There are two types of criteria commonly used for head impact injury evaluation: one is based on head kinematic and dynamic responses such as acceleration and velocity, and the other is based on brain tissue biomechanical responses. The former mainly includes the head injury criterion (HIC) (Administration, 1996), generalized acceleration model for brain injury threshold (GAMBIT) (Newman, 1986), rotational injury criterion (RIC) (Kimpara and Iwamoto, 2012), head impact power (HIP) (Newman and Shewchenko, 2000), and brain injury criterion (BrIC) (Takhounts et al., 2013). The latter mainly uses the cumulative strain damage measure (CSDM), intracranial pressure, maximum principal strain (MPS), and strain rate (Cloots et al., 2013; Wright and Ramesh, 2012). However, due to the existence of the resonance phenomenon, head impact injury may produce a response in some frequency bands with a certain vibration mode, resulting in the “amplification” of the deformation of brain injury (Fonville et al., 2022). For this case, the traditional head impact injury evaluation criteria based on the head response in the time domain make it difficult to comprehensively assess the mechanical response of the head under dynamic impact, and joint analysis of the response in the time and frequency domains is required to fully understand the characteristics of the head mechanical response signal.

The finite element (FE) modeling method, the reduced-order model method, and the skull–cerebrospinal fluid (CSF)–brain system fluid–solid coupling method are mainly used in studies of the frequency response of human body head mechanical response. El Baroudi and Razafimahery (2014), El Baroudi et al. (2012a), and El Baroudi et al. (2012b) developed a skull–cerebrospinal fluid–brain fluid–solid coupling model for modal analysis and found that the fundamental frequency of the head was 26.66 Hz. Laksari et al. (2015) proposed that the skull–brain dynamic can be approximated as an under-damped system and reported that the head exhibits significant resonant behavior at approximately 15 Hz ± 2.9 Hz (Laksari et al., 2015). Gabler et al. established a single-degree-of-freedom mechanical model to represent the brain–skull system and found that the intrinsic frequency of the brain was 22.3 Hz–27.5 Hz, which was close to the pulse duration (36 ms–45 ms) of the brain resonance period, and the shape of the brain displacement depends on the magnitude of velocity and acceleration (Ghodrati Amiri and Asadi, 2009). Yang et al. (2017) found that the fundamental frequency of the head finite element model was approximately 35.25 Hz for different damping factors. Laksari et al. (2018) reconstructed a mild traumatic brain injury case caused by collisions in American football players through dynamic mode decomposition (DMD) and finite element analysis, obtaining a fundamental frequency of 28 Hz (Laksari et al., 2018). Gabler et al. (2018) established a second-order detuned brain reduced-order model based on three-degree-of-freedom coupling and demonstrated that the inherent frequency of the brain–skull system was in the range of 21.6 Hz–29.3 Hz around the natural cycle of the brain. Recently, Fonville et al. (2022) extracted different free-end modes of the head FE model by eigenvalues and found that the fundamental frequency in the bound head mode was 22.3 Hz and the fundamental frequency in the free boundary brain tissue mode was 13.9 Hz (Fonville et al., 2022). However, most of the studies mentioned above focused on mild traumatic brain injury; the change in frequency-domain responses from mild to severe impact was not explored much. Furthermore, there is still a lack of combination analysis on frequency-domain response and traditional injury criteria.

The purpose of the current study is, therefore, to understand the characteristics of human body head frequency responses to blunt impacts and the correlation between head frequency responses and commonly used injury criteria. First, head blunt impacts under various boundary conditions were simulated using the FE modeling method. Then, the wavelet packet analysis method was employed to deal with the head acceleration signal for extracting frequency-domain characteristics and collecting energy. Finally, the characteristics of head frequency responses and their correlations with the commonly used kinematic- and biomechanical-based criteria were analyzed.

## 2 Materials and methods

### 2.1 FE simulation setup

**FIGURE 1:**
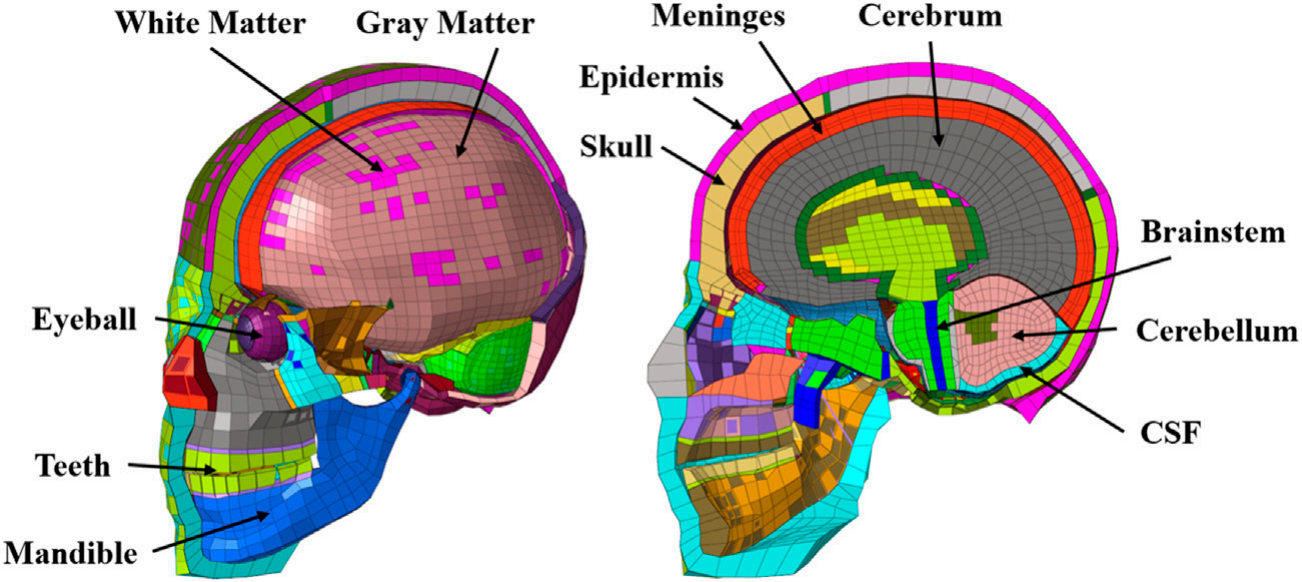
THUMS head model.

The head model extracted from the THUMS AM50 V4.0 human body model (mentioned as the THUMS head model below) was used for head blunt impact simulation. The THUMS head model consists of 37,756 nodes and 49,598 elements; the components include the skull, dura mater, brain, cerebellum, and brainstem, where the brain is surrounded by a layer of CSF, and the inner cranial bone consists of a hard shell to represent the anatomical structure of the head and brain more accurately (Figure 1). This model has been validated for its biofidelity (Iwamoto et al., 2002; Iwamoto et al., 2015; Kimpara et al., 2006; Watanabe et al., 2012) and is widely used in head blunt injury analysis (Iwamoto et al., 2002; Iwamoto et al., 2015; Wang et al., 2022; Wang et al., 2023); particularly, the THUMS head model showed good agreement with cadaver impact test data in terms of response force, acceleration, relative displacement between the intracranial tissue and skull, brain pressure, and biomechanical response (Iwamoto et al., 2002; Iwamoto et al., 2015).

**FIGURE 2:**
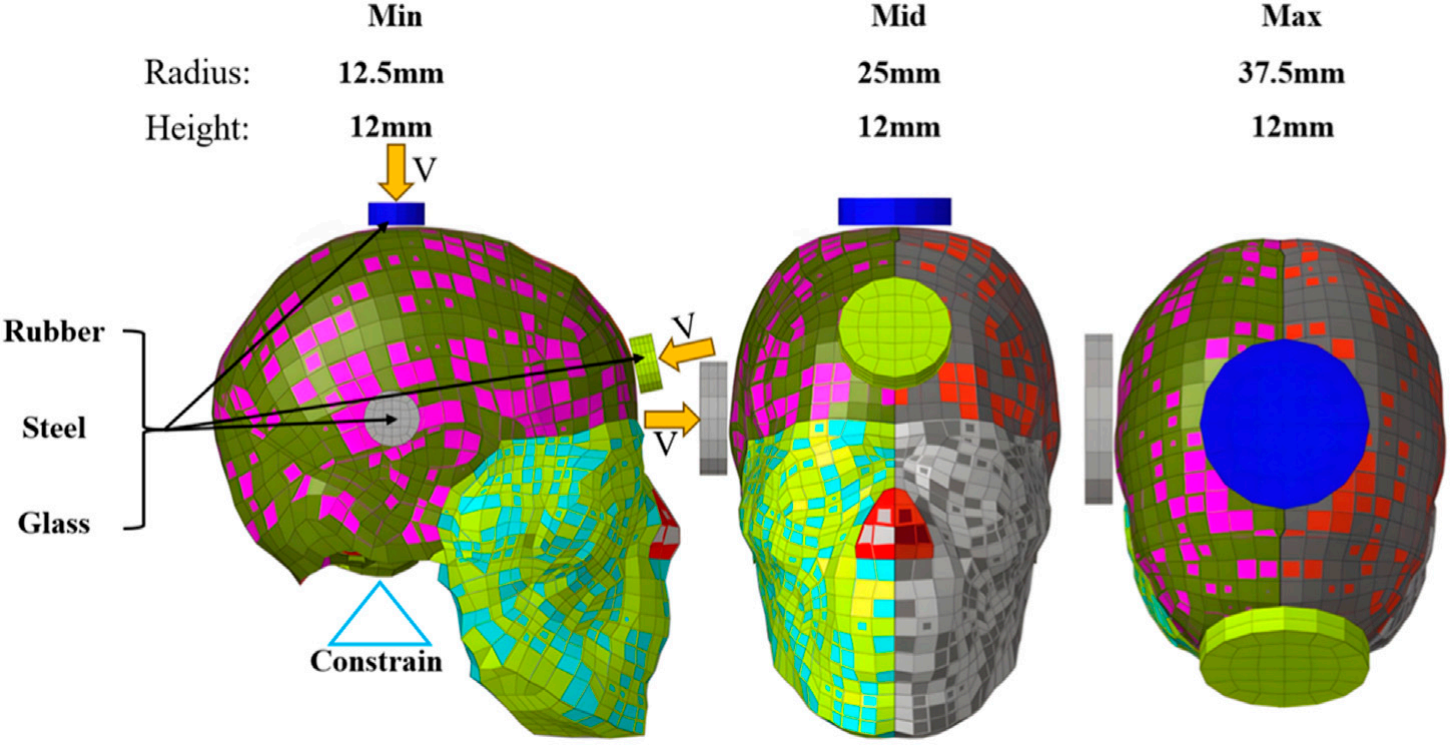
Impact block size, impact position, and material.

To simply simulate head blunt impacts under different boundary conditions, block-to-head impacts at various speeds were modeled using cylindrical impact blocks with different sizes and materials and the THUMS head model. A simulation matrix based on the full factorial test was defined using the parameters shown in Table 1. The detailed dimensions (min = 12.5 mm×12 mm, mid = 25 mm×12 mm, and max = 37.5 mm×12 mm) and impact locations (forehead center, right side near the wing point, and head top center) of the blocks are illustrated in Figure 2.

**TABLE 1:** Parameters for the definition of the simulation matrix.

Variable	Level		
Position	Right	Front	Top
Material	Rubber	Glass	Steel
Velocity	2 m/s	6 m/s	10 m/s
Size	Min	Mid	Max

In the simulations, a fixed constraint was applied to the surface of the occipital foramen of the THUMS head model to limit the head motion; a surface-to-surface contact with dynamic and static friction coefficients of 0.3 was defined between the skin and impact block outer surface; and the direction of the impact speed was set in the normal direction of the block. Three impact speeds were selected from low to high based primarily on common vehicle–pedestrian and falling object crash speeds (Li et al., 2021). Three impact block sizes were selected based on the contact area with the head for studying the influences of different blunt objects and different contact areas on the frequency-domain response of the head in real collision accidents. Three materials (rubber, steel, and glass) were selected, mainly according to the vehicle parts that occupants and vulnerable road users may contact in accidents, e.g., the interior upholstery, A-pillar, and windshield, which were modeled using different material models and properties (Table 2) and can represent structures with different stiffnesses. Three impact positions were chosen to investigate the influences of different skull thicknesses and distances from different brain tissues on the frequency-domain response of the head, which can basically represent the common impact positions of the head given the symmetrical structure of the head.

**TABLE 2:** Parameters of the impact blocks in different materials.

Material parameter	Rubber	Glass	Steel
Material model in LS-DYNA	ELASTIC	MODIFIED_PIECEWISE_LINEAR_PLASTICITY	PIECEWISE_LINEAR_PLASTICITY
Density (kg/mm^3^)	1.829e-06	2.5e-06	7.8e-06
Young’s modulus (Mpa)	4	70000	210000
Poisson’s ratio	0.45	0.23	0.3

### 2.2 Frequency response analysis

The wavelet packet transform (WPT) is one extension of the wavelet transform (WT) that provides complete level-by-level decomposition (Coifman and Wickerhauser, 1992). The WPT is commonly used to transform the sequence data on the time axis of non-stationary random signals (such as acceleration signals) into spectral data on time and frequency, which can provide information about the intensity of a non-stationary time-dependent motion of interest at a specific frequency, and it is easier to obtain the energy changes of each frequency band (Gabler et al., 2019). Furthermore, wavelet packets have a high frequency resolution for nonlinear signals and can segment the frequency band to study the contribution of different frequency bands to impact energy, which has the ability to link the frequency band to head injury. Therefore, the WPT was used to study the frequency-domain characteristics of non-stationary random signals of the head generated by blunt impacts.

The principle of wavelet packets can be illustrated as follows. Assuming that the wavelet packet function is $u_{n}\left(t\right)$ , it needs to satisfy the bi-scale equation

$$u_{2n}\left(t\right)=\sqrt{2}\sum_{k\in Z}h_{k}u_{n}\left(2t-k\right),$$ (1)

$$u_{2n+1}\left(t\right)=\sqrt{2}\sum_{k\in Z}g_{k}u_{n}\left(2t-k\right),$$ (2)

where *Z* is a positive integer; *h*_*k*_ and *g*_*k*_ are two-scale functions, *g*_*k*_ *=(-1)* ^*k*^ *h(1-k)*, which have an orthogonal relationship. The sequence {*u*_*n*_ *(t)*} constructed using Eqs 1, 2 is called the wavelet packet of the scaling function. When *n = 0*, *u*_*0*_ *(t)* and *u*_*1*_ *(t)* are the wavelet basis functions of the scale functions *φ(t)* and *ψ(t)*, respectively.

The wavelet packet decomposition program simply transforms the signal from time domain to frequency domain while maintaining equal energy (Lu et al., 2013). For an acceleration signal *x(t)* subjected to wavelet packet decomposition up to the i th level, the energy of each sub-band can be calculated using the following formula:

$$E_{ij}=\int {\left|S_{i,j}\right|}^{2}dt=\sum_{k=1}^{n}{\left|x_{i,j}\left(k\right)\right|}^{2},$$ (3)

and the formula for calculating the total energy is

$$E=\sum_{j=0}^{2^{i}-1}E_{ij}.$$ (4)

Appropriate wavelet basis is a key factor in the signal processing effect of wavelet packet analysis. Due to the fact that the skull–brain impact signal is a non-stationary random signal similar to seismic signals and mechanical fault signals, a previous study (Fonville et al., 2022) has shown that when the skull and brain are combined as a composite structure for analysis, the skull–brain frequency response characteristics are mainly characterized by low-frequency features and may exhibit certain abrupt signals. Therefore, an sym2 wavelet with a sampling frequency of 5000 Hz, which has tight support, poor regularity, low vanishing moment order, and orthogonality to maintain energy (Bianchi et al., 2015), was selected as the wavelet base in this paper. According to Shannon’s sampling theorem (Shannon, 2001), the Nyquist frequency is 2500 Hz. The number of wavelet packet decomposition layers was set to 9, which means that the original signal is divided into 512 sub-bands in the entire frequency domain, and each sub-band represents a bandwidth of 4.88 Hz.

### 2.3 Data analysis

The acceleration signals output from the simulations were subjected to wavelet packet-based frequency response analysis, which was extracted from the contralateral skull at the center of the head collision at a sampling frequency of 5000 Hz (i.e., sampling every 0.0002 s). This sampling frequency was defined by taking into account both the accuracy of the results and computing efficiency since a low sampling frequency may miss the key acceleration peaks and a high sampling rate may increase timing costs. Then, three potential head injury indicators in the frequency domain were calculated using Eqs 3, 4, which are *(E*_*ij*_ *)*_*max*_, *(i,j)*_*(Eij)max*_, and *(E*_*ij*_ */E*_*total*_ *)*_*max*_, denoting the energy of the sub-band with the maximum value over all sub-bands, the position of the sub-band with the maximum energy (multiplied by 4.88 Hz is the frequency band), and the proportion of *(E*_*ij*_ *)*_*max*_ in the whole energy, respectively. These three frequency-domain parameters can effectively extract key frequency-domain features contained in the signal: *(E*_*ij*_ *)*_*max*_ reflects the strength of the signal within that frequency band, *(i,j)*_*(Eij)max*_ indicates the frequency band that contains the maximum energy in the signal, and *(E*_*ij*_ */E*_*total*_ *)*_*max*_ denotes the concentration of frequency. Finally, the kinematic-based head injury criterion HIC and the biomechanical-based brain injury criterion MPS were also calculated for each simulation. Then, spectral analysis was conducted to understand the characteristics of head frequency responses; parametric analysis using the non-parametric test was performed to analyze the influences of impact boundary conditions on the outcome of *(E*_*ij*_ *)*_*max*_, *(i,j)*_*(Eij)max*_, *(E*_*ij*_ */E*_*total*_ *)*_*max*_, HIC, and MPS; and regression analysis based on linear fitting was carried out to study the correlation between frequency-domain head injury indicators and existing head/brain injury criteria HIC and MPS.

## 3 Results

### 3.1 Spectral analysis

**FIGURE 3:**
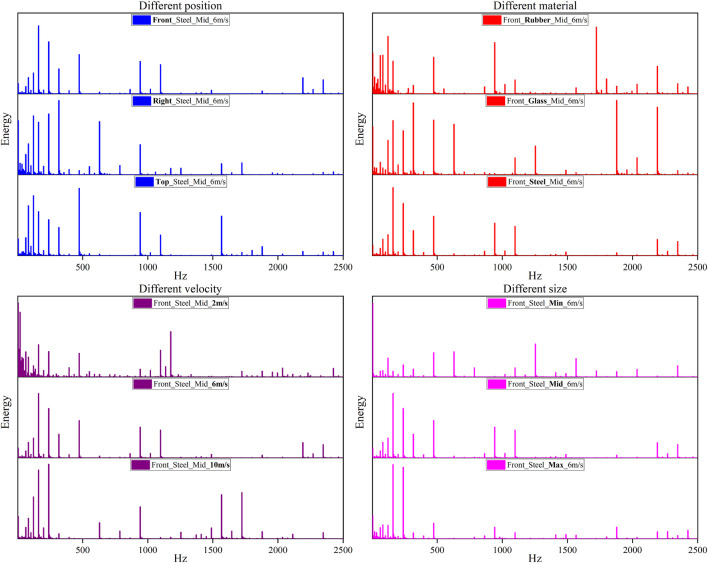
Typical energy–frequency response under different impact conditions.

Figure 3 shows the typical energy–frequency response of head acceleration signals from different impact conditions, where the case of Front_Steel_Mid_6 m/s was regarded as the reference condition and each sub-figure shows the variation of a parameter in Table 1 based on this reference condition. Generally, the head energy is distributed in a wide frequency range of 0–2500 Hz but mainly within the frequency bands 0–500 Hz. Furthermore, four energy concentration frequency regions can be observed, i.e., 0–35 Hz, 60–350 Hz, 450–500 Hz, and 900–1200 Hz. It is also found that there is no obvious difference in the energy distribution of each frequency band when changing the impact location; the head energy at the low-frequency bands (<100 Hz) decreases with the increase in the stiffness of the impact block from rubber to glass to steel and also the impact velocity; on the contrary, an opposite trend was observed for the change in impact block size, where the head energy is more concentrated in the low-frequency bands (<100 Hz) when impacted by a larger block.

**FIGURE 4:**
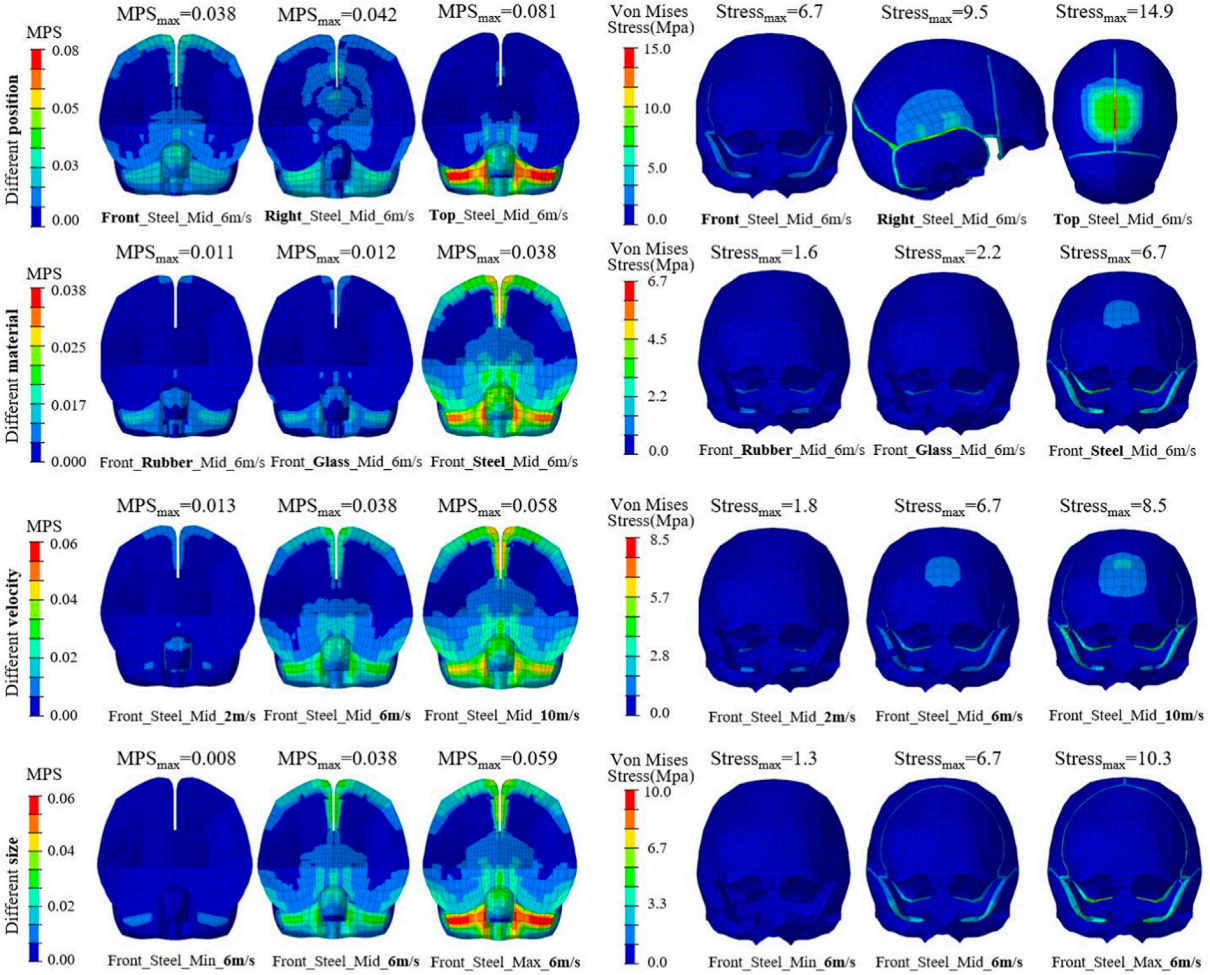
Typical distributions of brain MPS (left) and skull von Mises stress (right) under different impacts.

Figure 4 shows the distributions of brain MPS and skull von Mises stress under different impacts. Again, the case of Front_Steel_Mid_6 m/s was regarded as the reference, and the variations of all parameters were represented in example cases (the caption below each sub-figure illustrates the impact boundaries). It could be found that the brain strain mainly occurs in the peripheral areas of brain tissue, and the skull stress is mainly distributed in the impact block contact area and bone sutures. When changing the impact position from front to right to top, both MPS and von Mises stress gradually increase. The brain MPS and skull von Mises stress both increase with the increase in the stiffness, velocity, and size of the impact block.

### 3.2 Parametric analysis

**FIGURE 5:**
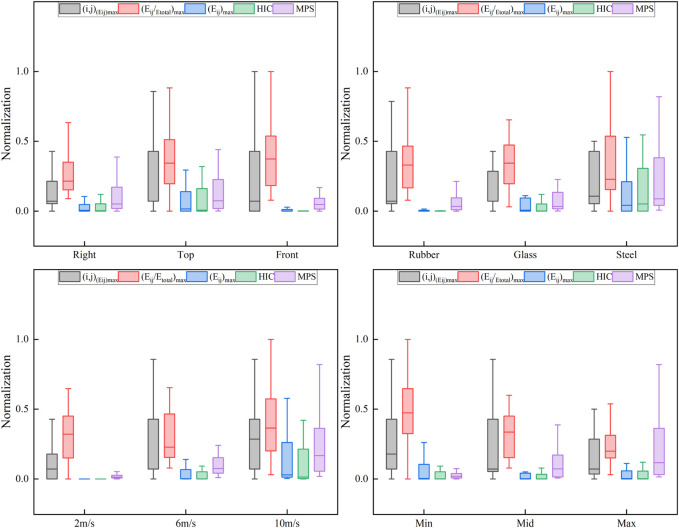
Distribution of the normalized values for the time-domain and frequency-domain head injury indicators as a function of impact position, impact block material, impact velocity, and impact block size.

Figure 5 shows the distribution of the normalized values for the time-domain and frequency-domain head injury indicators as a function of impact position, impact block material, impact velocity, and impact block size. For different impact positions, a wider fluctuation range and higher values of *(i,j)*_*(Eij)max*_ and *(E*_*ij*_ */E*_*total*_ *)*_*max*_ are observed in the top and front impact cases compared with the impacts at the right side, and the top impacts have the highest values of *(E*_*ij*_ *)*_*max*_, HIC, and MPS. When the material of the impact block shifts from rubber to glass to steel, both the fluctuation range and median value of the *(Eij)max*, HIC, and MPS increase, while the glass blocks lead to a generally lower *(i,j)*_*(Eij)max*_. A clear increasing trend was found for all indicators except *(E*_*ij*_ */E*_*total*_ *)*_*max*_ when increasing the impact velocity. Generally, a smaller block results in a higher value and fluctuation range of the *(i,j)*_*(Eij)max*_ and *(E*_*ij*_ */E*_*total*_ *)*_*max*_, while an opposite trend was found for the MPS. On the other hand, the middle-sized block induced a lower *(E*_*ij*_ *)*_*max*_ and HIC.

Table 3 shows the results of the non-parametric test, which was performed to evaluate the statistical significance of the influences of the impact boundary conditions on the selected time-domain and frequency-domain head injury indicators. It is found that the impact position has no significant effect on all head injury indicators. However, the material of the impact block has a significant influence on the *(E*_*ij*_ *)*_*max*_, HIC, and MPS; the size of the impact block significantly affects *(E*_*ij*_ */E*_*total*_ *)*_*max*_ and MPS values, while the impact velocity has a significant influence on all indicators except for *(E*_*ij*_ */E*_*total*_ *)*_*max*_.

**TABLE 3:** Non-parametric test results.

Item	*(i,j)*_*(Eij)max*_	*(E*_*ij*_ */E*_*total*_ *)*_*max*_	*(E*_*ij*_ *)*_*max*_	HIC	MPS
Position	0.26	0.15	0.18	0.09	0.49
Material	0.86	0.94	0.002*	0.002*	0.008*
Size	0.62	0.0000*	0.97	0.8	0.0000*
Velocity	0.01*	0.1	0.0000*	0.0000*	0.0000*

* A significant difference in the mean value at *p* < 0.05.

### 3.3 Correlation analysis

**FIGURE 6:**
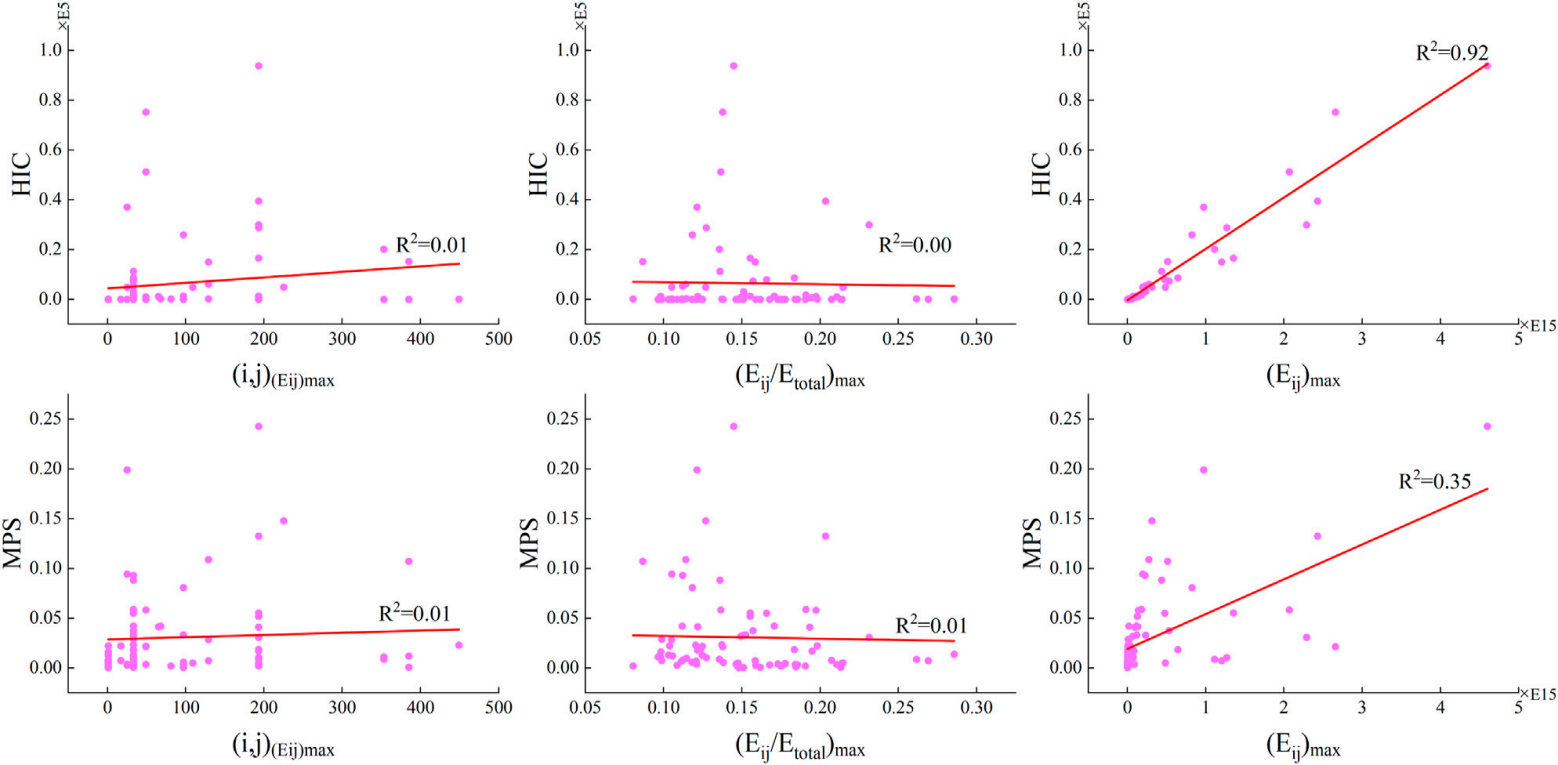
Linear fittings between the potential frequency-domain head injury indicators, *(i,j)*_*(Eij)max*_, *(E*_*ij*_ */E*_*total*_ *)*_*max*_, and *(E*_*ij*_ *)*_*max*_, and the HIC and MPS.

Figure 6 shows the linear fitting results between the potential frequency-domain head injury indicators, *(i,j)*_*(Eij)max*_, *(E*_*ij*_ */E*_*total*_ *)*_*max*_, and *(E*_*ij*_ *)*_*max*_, and the HIC and MPS. It can be clearly seen that *(E*_*ij*_ *)*_*max*_ has a high correlation with the HIC (with a linear fitting *R* ^2^ value of 0.92) and a weaker linear relationship with the MPS (*R* ^2^ = 0.35), whereas *(i,j)*_*(Eij)max*_ and *(E*_*ij*_ */E*_*total*_ *)*_*max*_ show almost no linear correlation with both HIC and MPS.

## 4 Discussion

In this study, the wavelet packet processing program was employed for investigating the characteristics of head frequency response under blunt impacts. The analysis results (Figure 3) indicate that the impact energy observed on the head is mainly in the frequency bands 0–500 Hz; for few cases with a low impact speed (2 m/s) or a softer block (rubber), high head energy can be seen around the head fundamental frequency (14–35 Hz) reported in the literature (El Baroudi et al., 2012a; El Baroudi et al., 2012b; El Baroudi and Razafimahery, 2014; Fonville et al., 2022; Ghodrati Amiri and Asadi, 2009; Laksari et al., 2015; Laksari et al., 2018; Yang et al., 2017); however, for most cases, the head energy is concentrated in the frequency regions much higher than the fundamental frequency of the head. This finding might suggest that resonance issues could not be considered in the head blunt impacts.

The simulation results (Figure 5; Table 3) also show that the frequency-domain response of head energy under blunt impacts is significantly sensitive to the impact velocity and the material and size of the impact block, except for the impact location. Particularly, a higher impact velocity usually leads to a higher head energy peak *(E*_*ij*_ *)*_*max*_ at a higher-frequency sub-band *(i,j)*_*(Eij)max*_, a stiffer impact block is associated with a higher head energy peak *(E*_*ij*_ *)*_*max*_, and a bigger impact block could result in a high proportion of the energy peak (over the total head energy) *(E*_*ij*_ */E*_*total*_ *)*_*max*_. It is easy to understand that higher impact velocity and a stiffer impact block (also higher density) could induce higher energy to the head, hence leading to a high energy peak *(E*_*ij*_ *)*_*max*_; this could also be verified by observing the variation trend in HIC and MPS while changing the impact velocity and material of the impact block. However, the material characteristics of the impact block might have a different influence on the frequency compared to the amplitude, and hence, the frequency of the energy peak *(i,j)*_*(Eij)max*_ is not sensitive to the stiffness of the impact block.

On the other hand, the proportion of the energy peak over the total head energy *(E*_*ij*_ */E*_*total*_ *)*_*max*_ is not affected by the impact boundary condition, given the fact that this parameter is affected by the value of both *(E*_*ij*_ *)*_*max*_ and *E*_*total*_, which usually accompany an increase or decrease (a higher *(E*_*ij*_ *)*_*max*_ is usually in case with a higher *E*_*total*_). The impact position has no significant effect on head energy; this might be mainly because of the integrated structure of the skull, where different impact positions do not affect the absorption and propagation of impact energy. It is surprising that the size of the impact block has no significant effect on the head energy peak *(E*_*ij*_ *)*_*max*_. This may be due to the fact that as the size (and mass) of the impact block increases, the contact area between the head and the impact block also increases, resulting in more energy absorption and less pressure on the skull. However, a bigger impact block could induce more intense movements in the brain, resulting in a higher MPS.

Furthermore, the linear fitting results (Figure 6) between the frequency-domain parameters *(i,j)*_*(Eij)max*_, *(E*_*ij*_ */E*_*total*_ *)*_*max*_, and *(E*_*ij*_ *)*_*max*_ and existing head injury criteria HIC and MPS indicate that only *(E*_*ij*_ *)*_*max*_ has a linear correlation with HIC (*R* ^2^ = 0.92) and MPS (*R* ^2^ = 0.35). This may suggest that the head energy peak *(E*_*ij*_ *)*_*max*_ has a certain contribution to head impact injury, particularly to skull fracture, as HIC was initially developed based on skull fracture risk (Coifman and Wickerhauser, 1992). The high linear correlation between *(E*_*ij*_ *)*_*max*_ and HIC could also be easily understood by the fact that they are both based on the head acceleration signal. This finding may suggest that only the amplitude–frequency aggregation characteristic of the head acceleration response has head impact injury predictability under blunt impacts, while the frequency itself does not seem to be related to head injuries.

There are several limitations to the current work. First, the blunt impacts simulated in the current study were simplified as pure linear load; rotation may induce a significant influence, and future analysis may focus on real crash scenarios to further understand head frequency response. Second, the frequency response of THUMS has not been validated against biomechanical test data, although this model showed good injury-predictive capability in the literature. Finally, the defined frequency-domain injury predictors show a low correlation with the MPS, and other frequency-domain parameters would be focused on in further analysis.

## 5 Conclusion

The current study is the first attempt to understand the characteristics of head frequency responses in blunt impacts of varying severity, where the FE human body models, wavelet packet frequency response analysis method, and statistical analysis method were employed. The simulation results show that the frequency-domain responses of head energy in blunt impacts could be affected by the impact boundary condition. The head energy peak and its frequency increase with the increase in impact; a stiffer impact block is associated with a higher head energy peak, and a bigger impact block could result in a high proportion of the energy peak. Regression analysis indicates that only the head energy peak has a high correlation with exiting head injury criteria, which implies that the amplitude–frequency aggregation characteristic but not the frequency itself of the head acceleration response has predictability for head impact injury in blunt impacts. The findings of the current study may provide additional criteria for head impact injury evaluation and new ideas for head impact injury protection.

## Data Availability

The raw data supporting the conclusion of this article will be made available by the authors, without undue reservation.

## References

[B1] Administration N. H. T. S. (1996). Federal motor vehicle safety standards; Occupant crash protection; Final rule.61. Fed. Regist. 230 (27), 60206–60221.

[B2] Bianchi D. Mayrhofer E. Gröschl M. Betz G. Vernes A. (2015). Wavelet packet transform for detection of single events in acoustic emission signals. Mech. Syst. Signal P. R. 64-65, 441–451. 10.1016/j.ymssp.2015.04.014

[B3] Cloots R. J. van Dommelen J. A. Kleiven S. Geers M. G. (2013). Multi-scale mechanics of traumatic brain injury: predicting axonal strains from head loads. Biomech. Model Mechanobiol. 12 (1), 137–150. 10.1007/s10237-012-0387-6 22434184

[B4] Coifman R. R. Wickerhauser M. V. (1992). Entropy-based algorithms for best basis selection. IEEE T Inf. Theory 38 (2), 713–718. 10.1109/18.119732

[B5] El Baroudi A. Razafimahery F. (2014). Theoretical and numerical investigations of frequency analysis of two circular cylinders oscillating in a incompressible viscous fluid. Int. J. Appl. Mech. 06 (05), 1450049. 10.1142/s1758825114500495

[B6] El Baroudi A. Razafimahery F. Rakotomanana L. R. (2012b). Parametric modal analysis of the brain-CSF-skull system. Eur. J. Comput. Mech. 18 (1), 55–66. 10.13052/ejcm.18.55-66

[B7] El Baroudi A. Razafimahery F. Rakotomanana-Ravelonarivo L. (2012a). Three-dimensional modal analysis of an idealized human head including fluid–structure interaction effects. Acta Mech. 223 (9), 1899–1915. 10.1007/s00707-012-0681-5

[B8] Fonville T. R. Scarola S. J. Hammi Y. Prabhu R. K. Horstemeyer M. F. (2022). “Resonant frequencies of a human brain, skull, and head,” in Multiscale biomechanical modeling of the brain (Pittsburgh, America), 239–254.

[B9] Gabler L. F. Crandall J. R. Panzer M. B. (2019). Development of a second-order system for rapid estimation of maximum brain strain. Ann. Biomed. Eng. 47 (9), 1971–1981. 10.1007/s10439-018-02179-9 30515603

[B10] Gabler L. F. Joodaki H. Crandall J. R. Panzer M. B. (2018). Development of a single-degree-of-freedom mechanical model for predicting strain-based brain injury responses. J. Biomech. Eng. 140 (3), 031002. 10.1115/1.4038357 29114772

[B11] Ghodrati Amiri G. Asadi A. (2009). Comparison of different methods of wavelet and wavelet packet transform in processing ground motion records. Int. J. Civ. Eng. 7 (4), 248–257.

[B12] Iwamoto M. Kisanuki Y. Watanabe I. Furusu K. Hasegawa J. (2002). Development of a finite element model of the total human model for safety (THUMS) and application to injury reconstruction. In: proceedings of the international IRCOBI conference. Munich, Germany.

[B13] Iwamoto M. Nakahira Y. Kimpara H. (2015). Development and validation of the total human model for safety (THUMS) toward further understanding of occupant injury mechanisms in precrash and during crash. Traffic Inj. Prev. 16 (1), S36–S48. 10.1080/15389588.2015.1015000 26027974

[B14] Kimpara H. Iwamoto M. (2012). Mild traumatic brain injury predictors based on angular accelerations during impacts. Ann. Biomed. Eng. 40 (1), 114–126. 10.1007/s10439-011-0414-2 21994065

[B15] Kimpara H. Nakahira Y. Iwamoto M. Miki K. Taguchi T. J. S. C. C. J. Kawano S. i. (2006). Investigation of anteroposterior head-neck responses during severe frontal impacts using a brain-spinal cord complex FE model. Stapp Car Crash J. 50 (2), 509–544. 10.4271/2006-22-0019 17311175

[B16] Laksari K. Kurt M. Babaee H. Kleiven S. Camarillo D. (2018). Mechanistic insights into human brain impact dynamics through modal analysis. Phys. Rev. Lett. 120 (13), 138101. 10.1103/physrevlett.120.138101 29694192

[B17] Laksari K. Wu L. C. Kurt M. Kuo C. Camarillo D. C. (2015). Resonance of human brain under head acceleration. J. R. Soc. Interface 12 (108), 20150331. 10.1098/rsif.2015.0331 26063824 PMC4528602

[B18] Li G. Liu J. Li K. Zhao H. Shi L. Zhang S. (2021). Realistic reference for evaluation of vehicle safety focusing on pedestrian head protection observed from kinematic reconstruction of real-world collisions. Front. Bioeng. Biotechnol. 9, 768994. 10.3389/fbioe.2021.768994 34993187 PMC8724547

[B19] Lu J. Wang B. Liang D. (2013). Wavelet packet energy characterization of low velocity impacts and load localization by optical fiber Bragg grating sensor technique. Appl. Opt. 52 (11), 2346–2352. 10.1364/ao.52.002346 23670766

[B20] MacDonald C. L. Johnson A. M. Nelson E. C. Werner N. J. Fang R. Flaherty S. F. (2014). Functional status after blast-plus-impact complex concussive traumatic brain injury in evacuated United States military personnel. J. Neurotrauma 31 (10), 889–898. 10.1089/neu.2013.3173 24367929 PMC4012688

[B21] Newman J. (1986). “A generalized acceleration model for brain injury threshold,” in International research council on Biomechanics of injury(IRCOBI) conference (Zurich, Switzerland).

[B22] Newman J. A. Shewchenko N. (2000). A proposed new biomechanical head injury assessment function-the maximum power index. SAE Technical Paper No. 2000-01-SC16. 10.4271/2000-01-SC16 17458729

[B23] Organization W. H. (2018). Global status report on road safety 2018. Geneva, Switzerland: Summary.

[B24] Popescu C A. A. Daia C. Onose G. (2015). Actual data on epidemiological evolution and prevention endeavours regarding traumatic brain injury. J. Med. Life 8 (3), 272–277. 26351526 PMC4556905

[B25] Shannon C. E. (2001). A mathematical theory of communication. ACM Sigmob. Mob. Comput. Commun. Rev. 5 (1), 3–55. 10.1145/584091.584093

[B26] Takhounts E. G. Craig M. J. Moorhouse K. McFadden J. Hasija V. (2013). Development of brain injury criteria (BrIC). Stapp Car Crash J. 57, 243–266. 10.4271/2013-22-0010 24435734

[B27] Wang F. Peng K. Zou T. Li Q. Li F. Wang X. (2023). Numerical reconstruction of cyclist impact accidents: can helmets protect the head-neck of cyclists? Biomimetics 8 (6), 456. 10.3390/biomimetics8060456 37887587 PMC10603864

[B28] Wang F. Wu J. Hu L. Yu C. Wang B. Miller K. (2022). Evaluation of the head protection effectiveness of cyclist helmets using full-scale computational biomechanics modelling of cycling accidents. J. Saf. Res. 80, 109–134. 10.1016/j.jsr.2021.11.005 35249593

[B29] Watanabe R. Miyazaki H. Kitagawa Y. Yasuki T. J. A. R. J. (2012). Research of collision speed dependency of pedestrian head and chest injuries using human FE Model (THUMS Version 4). Accid. Reconstr. J. 22 (1), 31–40.

[B30] Wright R. M. Ramesh K. T. (2012). An axonal strain injury criterion for traumatic brain injury. Biomech. Model Mechanobiol. 11 (1-2), 245–260. 10.1007/s10237-011-0307-1 21476072

[B31] Yang B. Shi Z. Wang Q. Xiao F. Gu T. Yin Y. K. (2017). Frequency spectrum of the human head–neck to mechanical vibrations. J. Low. Freq. Noise V. A 37 (3), 611–618. 10.1177/1461348417747179

